# Supplementary material for: Standing-wave-excited multiplanar fluorescence in a laser scanning microscope reveals 3D information on red blood cells
Source: Sci Rep. 2014 Dec 8;4:7359. doi: 10.1038/srep07359 (PMC4258645; doi:10.1038/srep07359)
Supplement: Supplementary Information — for “Standing-wave-excited multiplanar fluorescence in a laser scanning microscope reveals 3D information on red blood cells” [file srep07359-s1.pdf]

Supplementary Information for “Standing-wave-excited multiplanar fluorescence in a laser-scanning microscope reveals 3D information on red blood cells”

Rumelo Amor, Sumeet Mahajan, William Bradshaw Amos, and Gail McConnell

(Dated: 26 September 2014)

S1. Experimental setup

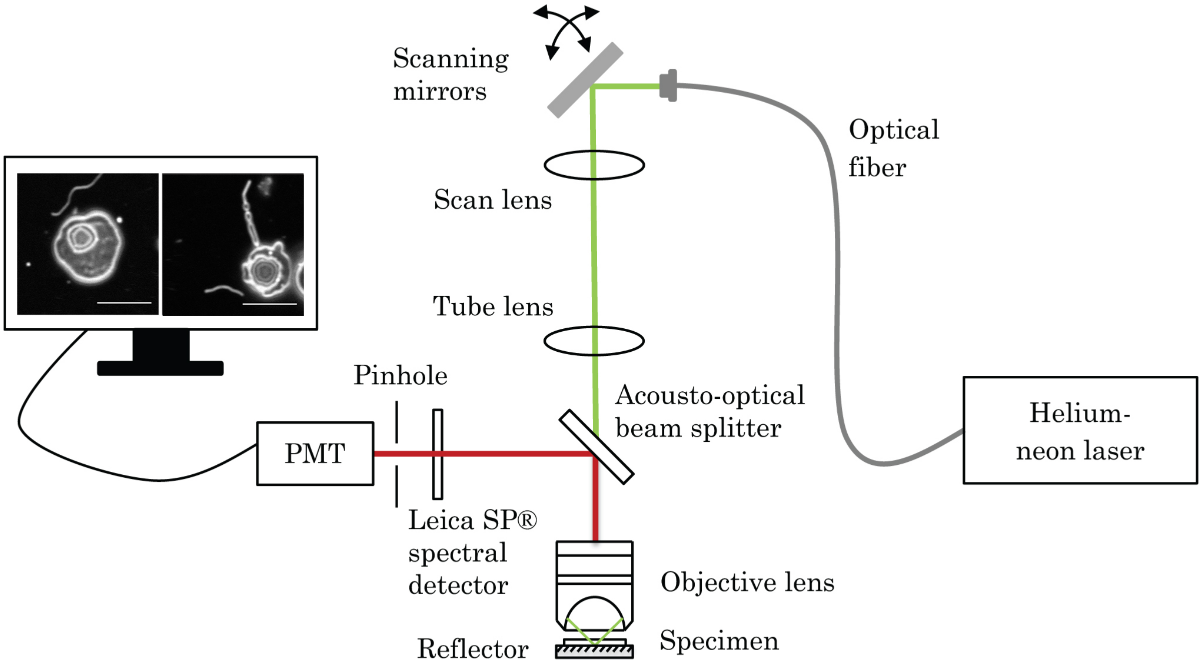

FIG. S1. Schematic diagram of the setup for standing-wave excitation of fluorescence in a laser-scanning microscope, shown here specifically for imaging mouse red blood cell ghosts. The ghosts were stained with the membrane-specific dye DiI, mounted on top of a mirror and imaged with a Leica SP5 confocal microscope using a 100x/1.40 N.A. oil immersion objective. The excitation wavelength used was 543 nm and the fluorescence emission was detected at 555–655 nm using the Leica SP® spectral detector. The detector uses a prism to disperse light emitted from the specimen and on its way to the sensor, the spectrum is made to pass through a steplessly adjustable slit photometer device made up of two motorized barriers that can be moved to any wavelength position and has a spectral resolution of 5 nm[1–3]. Interference between the incoming excitation wave and its reflection from the mirror creates a standing wave. Since the ghost cell membrane is of biconcave shape, a pattern of fringes is produced as a result of fluorescence excitation at the standing-wave antinodes.

S2. Origin of apparently planar antinodal zones even with objectives of high numerical aperture in a single-point scanning microscope

In standing-wave microscopy using wide-field illumination, two nearly-collimated Gaussian laser beams are made to interfere using a reflector, creating a standing wave with nodal surfaces that are flat to better than 1 part in 20,000 over the field of view and therefore can be considered as planes parallel to the reflector[4]. Here we discuss the origin of apparently planar antinodal zones even with objectives of high numerical aperture in a single-point scanning microscope, where the focussed spot produces curved antinodes.

When a standing wave is formed by reflection from a mirror as in Fig. S2a, the mirror surface is a node, where no radiation can be detected[5–7]. There are more nodes at intervals of half a wavelength and between the nodes, there are antinodes of high radiation intensity. The first antinode is a quarter of a wavelength away from the mirror and succeeding antinodes are at half-wavelength intervals. Spherical waves converging on a mirror surface AB will interfere with the reflected diverging spherical waves, creating nodes and antinodes as shown in Fig. S2b. In the figure, we have taken into account the evolution of the wavefront radius of curvature with distance from the beam waist, hence the first antinode has a noticeably different radius of curvature. The fundamental Gaussian amplitude field, which is a solution the beam wave equation[8], shows that as the beam comes in to a focus at the mirror surface, the phase fronts are spherical with contracting radius of curvature. However, the radius of curvature reaches a minimum just before the focus then diverges so that the wavefront at the beam waist is planar. For an oil-immersion objective with a numerical aperture of 1.30 and using $\lambda = 488$ nm and $n = 1.52$, such as what was used in Fig. 1b, this minimum occurs 210 nm away from the mirror surface. Therefore, beyond 210 nm, where all the antinodes except the first are located, the antinodes are curved. Along all radii stretching out from the point of focus, the wave train is a perfect standing wave, but each node and antinode is a spherical shell. If a single-point scanning microscope were used and a small (sub-wavelength) fluorescent object were moved along the vertical axis away from the focus and towards the lens, we would expect to see strong maxima when it passed through the first few antinodes. Off-axis fluorescent objects would also be excited by the spherical antinodes but these would not be detected efficiently.

Figure S2c shows the effect of the mirror AB being located one wavelength away from

the focus. This creates a virtual image of the focus two wavelengths (four node intervals) away from the focus. Along the line CE, there is a standing wave train, like one of the radii in Fig. S2b, but this is true only along CE and for directions at small angles to CE, that is, in the narrow sector where the antinodes are shown in blue. To take an extreme case, along CD, the phase front spacing is changing for the wave train originating in the mirror but is constant for the incoming wave train (supposing that the numerical aperture of the lens was sufficient to send in waves at such a high angle to the axis) and so the superposition of the incoming beam and its reflection produces no standing wave and there are no minima constant in position. This is true for most of the directions between CD and the blue sector. Since the illumination would have no minima in the wave trains at high angles, it would not discriminate depth. This explains why objectives of high numerical aperture fail to pick up high-order fringes, where the scanning spot is being reflected from a distant mirror such as in this case: the minima would be lost and a high background brightness would occur beyond the first few fringes, as is indeed observed. For the fringe pattern in Fig. 1d, which was taken with a 40x/1.30 N.A. objective, the contrast deteriorates rapidly from 93 % for the first fringe to 14 % for the fourth fringe. On the other hand, an objective of low numerical aperture could generate and detect only the wave trains in the blue sector and would ultimately approximate to a parallel-beam interferometer, capable of detecting fringes of very high order, as in Fig. 1c. Using the fundamental Gaussian amplitude field $U(r, z)$ in Ref. 8, and taking $U_1 = U(r, z)$ for the incoming beam, $U_2 = -U(r, -(z - 2\Delta z))$ for the reflection from a mirror defocused by Δz, $U_S = U_1 + U_2$ is the superposition of the incoming and reflected beams and $U_S U_S^*$ gives the standing-wave field structure. For the 5x/0.15 N.A. objective used to obtain Fig. 1c, the plot of $U_S U_S^*$ versus z in Fig. S2d shows that indeed, fringes of very high order are detected, with no appreciable change in fringe contrast, being 100 % for the 1st fringe and 99 % for the 37th fringe. Figure S2d agrees with the axial plot of fluorescence intensity in Fig. 2c.

Therefore, the nodes and antinodes in single-point laser-scanning are spherical when the mirror is close to the focus but are reduced to progressively smaller disks centred on the axis as the mirror moves away from the focus. The spherical shells appear planar because the relatively small emission in the disks is assigned to the pixel at the centre as scanning takes place.

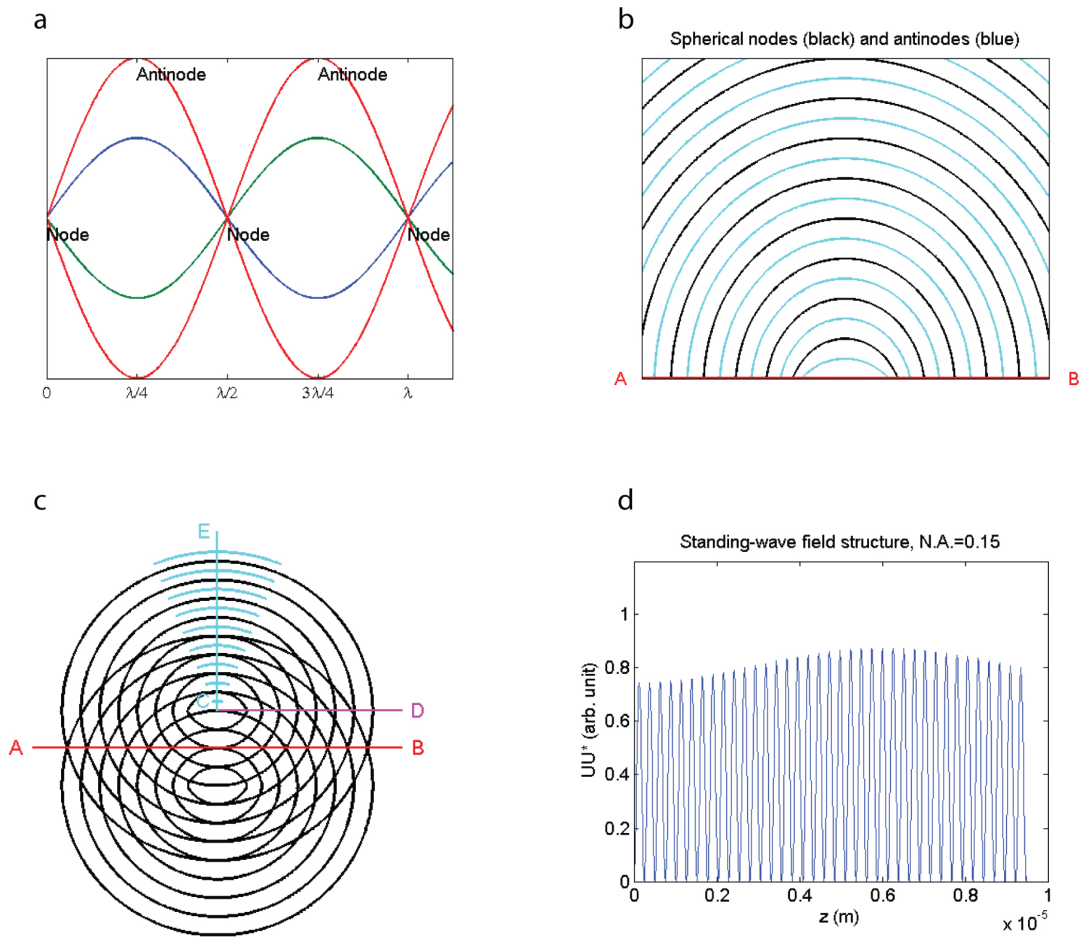

FIG. S2. Detection of planar standing-wave antinodes in single-point laser-scanning. (*a*) Standing-wave formation by reflection from a mirror, showing nodes and antinodes. (*b*) Spherical waves converging on a mirror surface AB interfere with their reflection, creating spherical nodes (black) and antinodes (blue). (*c*) The mirror AB located one wavelength away from the focus creates a virtual image of the focus two wavelengths away. Along the line CE and at small angles to CE, there is a standing wave train (blue sector). Along CD, and for most of the directions between CD and the blue sector, the phase front spacing is changing for the wave train from the mirror but is constant for the incoming wave train, therefore the superposition of the incoming beam and its reflection produces no standing wave. The spherical shells along CE appear planar because the small emission in the disks is assigned to the central pixel as scanning takes place. (*d*) Standing-wave field structure for a 5x/0.15 N.A. objective, showing fringes of very high order can be detected using a low-N.A. objective, with no appreciable change in fringe contrast, being 100 % for the 1st fringe and 99 % for the 37th fringe. The field intensity is in agreement with the axial plot of fluorescence intensity in Fig. 2c.

S3. Testing whether the fringes are caused by a standing wave or by a Fabry-Perot cavity effect

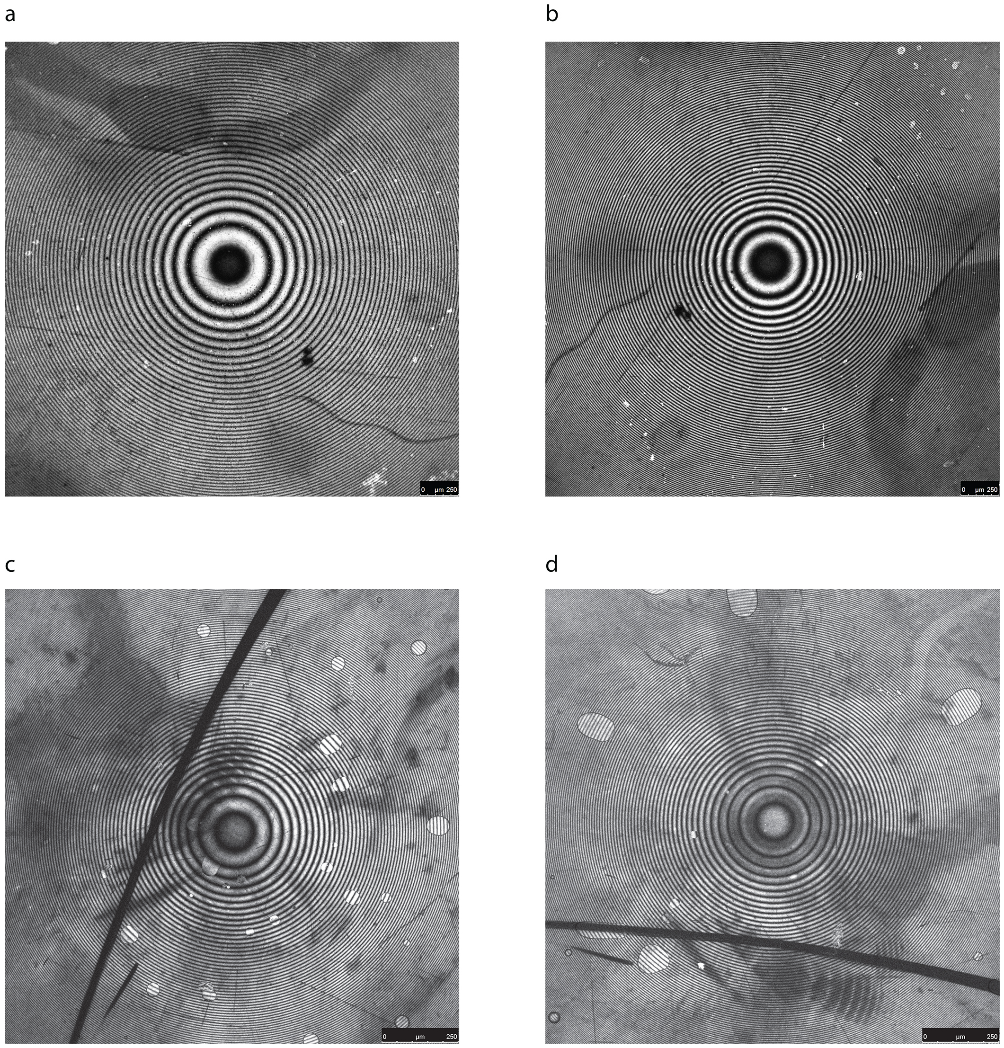

FIG. S3. In order to test whether the fluorescence fringes were caused by standing-wave excitation formed by the interaction of the excitation light with the mirror, and not by a Fabry-Perot cavity effect, where the cavity was formed by the silica-air interface on one side and the mirror on the other, we constructed specimens in which we had (*a*) air (n=1), (*b*) immersion oil (n=1.52)[9], (*c*) pure glycerol (n=1.47)[10], and (*d*) a solution of 91 % glycerol in water (n=1.46)[10] between the substrate and the mirror. Putting a film of fluid that has the same refractive as the silica lens (n=1.46)[11] eliminates reflection from the convex surface. We observed the concentric fluorescence fringes in all cases, proving that the fringe pattern is caused by a standing wave only. Scale bar=250 μm.

S4. Assessing the shape of red blood cells using differential interference contrast (DIC) microscopy

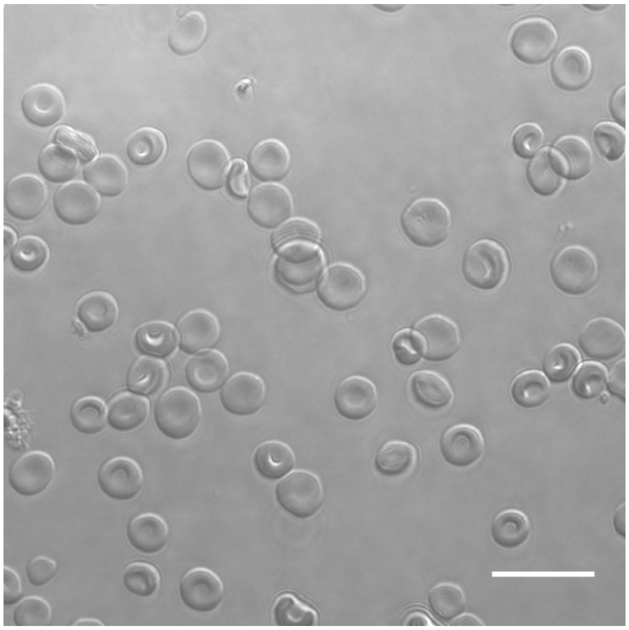

FIG. S4. DIC image of mouse red blood cell specimens used in Fig. 4, showing that the cells are normal discocytes. Scale bar=15 μm.

REFERENCES

[1] Engelhardt, J. World Patent WO 95/07447 (1995).

[2] Leica Spectral Detector SP® Flyer (2009). Available at www.leica-microsystems.com. Date of access: 10/09/2014.

[3] Leica TCS SP5 II Technical Documentation (2009). Available at www.leica-microsystems.com. Date of access: 16/09/2014.

[4] Bailey, B., Farkas, D.L., Taylor, D.L. & Lanni, F. Enhancement of axial resolution in fluorescence microscopy by standing-wave excitation. *Nature* **366,** 44–48 (1993).

[5] Wiener, O. Stehende lichtwellen und die schwingungsrichtung polarisirten lichtes. *Ann. Phys.* **276,** 203–243 (1890).

[6] Hertz, H. Ueber die Ausbreitungsgeschwindigkeit der electrodynamischen Wirkungen. *Ann. Phys.* **270,** 551–569 (1888).

[7]Hecht, E. *Optics*, 3rd ed (Addison Wesley Longman, Inc., Reading, Massachusetts, 1998).

[8]Kogelnik, H. & Li, T. Laser beams and resonators. *Appl. Opt.* **5,** 1550–1567 (1966).

[9]Immersion Media for Objectives (2011). Available at www.leica-microsystems.com. Date of access: 25/04/2014.

[10]Density of Glycerine-Water Solutions (2013). Available at www.dow.com. Date of access: 25/02/2014.

[11]Optical Materials Technical Information (2013). Available at www.comaroptics.com. Date of access: 25/04/2014.
